# SREBF1 mediates immunoparalysis of dendritic cells in sepsis by regulating lipid metabolism and endoplasmic reticulum stress

**DOI:** 10.1186/s12964-025-02295-9

**Published:** 2025-06-16

**Authors:** Yaolu Zhang, Fangfang Wu, Yan Li, Jiaxin Liu, Liuyan Zhu, Min Zhang, Zhongqiu Lu

**Affiliations:** 1 Emergency Department, The First Affiliated Hospital of Wenzhou Medical University, Ouhai District, Wenzhou, 325000 China; 2 Wenzhou Key Laboratory of Emergency and Disaster Medicine, Wenzhou, 325000 China

**Keywords:** SREBF1, Sepsis, Dendritic cells, Lipid metabolism, Endoplasmic reticulum stress

## Abstract

**Background:**

Lipid metabolic reprogramming is a key feature of sepsis, with increased lipid storage contributing to disease progression. Although lipid metabolism dysregulation has been implicated in sepsis pathogenesis, how lipid biosynthesis, particularly mediated by sterol regulatory element-binding transcription factor 1 (SREBF1), leads to dendritic cell (DC) immunoparalysis remains unclear.

**Methods:**

Intracellular lipid accumulation was assessed by Oil Red O and BODIPY staining. Gene and protein expression levels were analyzed via qPCR, Western blot, and immunofluorescence. SREBF1 activity was modulated using genetic knockout and siRNA silencing. DC phenotype and CD4^+^ T cell proliferation were evaluated using flow cytometry and co-culture assays. Cytokine secretion was measured using ELISA.

**Results:**

In a cecal ligation and puncture-induced sepsis model, we observed increased lipid biosynthesis and significantly elevated SREBF1 expression in spleen DCs. Increased SREBF1 expression suppressed the expression of costimulatory molecules (e.g., CD40, CD80, and CD86) and MHC II, reduced the secretion of inflammatory cytokines (e.g., TNFα, IL-1β, IL-6, and IL-12), impaired CD4^+^ T cell activation, and promoted apoptosis. Mechanistically, SREBF1 activation enhanced lipid biosynthesis in DCs, which triggered endoplasmic reticulum (ER) stress, as evidenced by increased PERK phosphorylation, eIF2α activation, and subsequent ATF4/CHOP induction. SREBF1 silencing attenuated the lipid-induced ER stress and restored DC function, whereas tunicamycin treatment partially reversed these protective effects.

**Conclusions:**

Our study identifies SREBF1 as a central regulator of sepsis-induced DC immunoparalysis by coupling lipid metabolic reprogramming to ER stress activation. Targeting this SREBF1-lipid-ER stress axis represents a novel strategy to reverse immunosuppression in septic patients.

**Supplementary Information:**

The online version contains supplementary material available at 10.1186/s12964-025-02295-9.

## Graphical Abstract

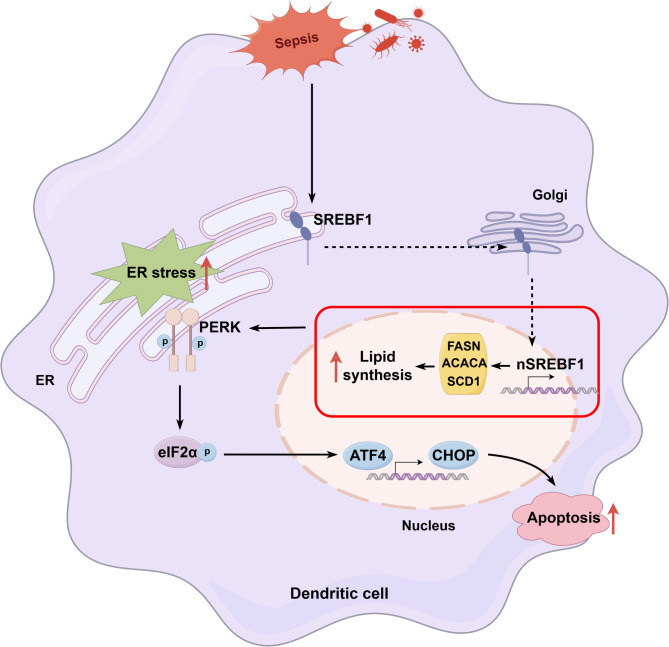

## Introduction

Sepsis is widely recognized as a severe organ dysfunction resulting from a dysregulated host response to infection [1]. This condition triggers a complex immune response involving simultaneous activation of pro-inflammatory and anti-inflammatory pathways. Consequently, many sepsis patients rapidly develop severe immunosuppression [2]. Sepsis-induced immunosuppression arises from impairments in both innate and adaptive immunity, including the release of anti-inflammatory cytokines, lymphocyte exhaustion, and the reprogramming of immune cell functions [3, 4]. This immunosuppression predisposes patients to secondary infections and long-term immune dysfunction, ultimately leading to increased late-stage mortality [5]. Specifically, among patients who survive the first 30 days following sepsis admission, over 40% die within the subsequent two years [6]. As the most efficient antigen-presenting cells (APCs), dendritic cells (DCs) play a pivotal role in linking innate and adaptive immunity [7]. Prior studies have consistently demonstrated that DCs are involved in immune dysregulation in sepsis, indicating their potential as therapeutic targets for immune modulation [8, 9]. However, the mechanisms underlying the DC-mediated immunoparalysis induced by sepsis are not well understood.

Emerging evidence has highlighted that metabolic reprogramming, particularly of lipid biosynthesis, is essential for DC development, survival, and immune function [10–12]. In particular, lipid biosynthesis has been shown to shape DC development and modulate their immunological activity. For instance, hepatic DCs with elevated lipid content display enhanced immunogenicity rather than promoting tolerance [13]. Conversely, intracellular lipid accumulation in DCs impairs their ability to effectively stimulate allogeneic T cells or efficiently present antigens, ultimately compromising immune responses [14, 15]. These findings underscore the profound impact of lipid metabolism on DC function. Despite advances in understanding lipid metabolic reprogramming in DCs, its specific role and underlying mechanisms in sepsis-induced immunosuppression remain unclear. Therefore, elucidating how lipid metabolism regulates DC activation in sepsis is essential for understanding immune dysfunction and holds significant therapeutic potential.

Sterol regulatory element-binding proteins (SREBPs) directly regulate the expression of over 30 genes involved in the biosynthesis and uptake of cholesterol, fatty acids, triglycerides, and phospholipids [16]. Among the two mammalian SREBP isoforms, SREBF1 primarily promotes the transcription of genes involved in fatty acid synthesis, whereas SREBF2 is more focused on activating cholesterol biosynthesis [17]. SREBF1 has emerged as a critical integrator of lipid metabolism and immune function. Mice with targeted deletion of SREBF1a exhibit resistance to endotoxin shock and the systemic inflammatory response syndrome induced by cecal ligation and puncture (CLP) [18]. Mechanistic studies reveal that SREBF1 enhances IL-1β production by upregulating NLRP3 gene expression in response to LPS in macrophages. Moreover, SREBF1 is activated by the T helper 2 (Th2) cell cytokine IL-4 and initiates de novo lipogenesis (DNL), a key process in macrophage alternative activation [19]. These observations suggest that SREBF1 serves as a key metabolic checkpoint in immune cells, linking lipid metabolism to functional outcomes. Given the central role of DCs in bridging innate and adaptive immunity, we hypothesize that SREBF1-mediated lipid metabolism similarly plays a regulatory role in DC function. Using a murine sepsis model and bone marrow-derived DCs (BMDCs), we demonstrate that upregulation of SREBF1 led to lipid overload, which subsequently induced endoplasmic reticulum (ER) stress. This lipid-driven ER stress was mediated through the PERK/eIF2α/ATF4/CHOP signaling pathway and contributed to DC immunoparalysis and apoptosis. Our findings unveil SREBF1 as a novel therapeutic target to reverse immunosuppression in sepsis.

## Materials and methods

### Ethics approval

This study was approved by the Ethics Committee of the First Affiliated Hospital of Wenzhou Medical University (Approval No. KY2024-R191). Written informed consent was obtained from all participants or their legal guardians prior to sample collection and data analysis. All procedures involving human participants were conducted in accordance with the ethical standards of the Declaration of Helsinki.

All animal experiments were approved by the Institutional Animal Care and Use Committee of the First Affiliated Hospital of the Wenzhou Medical University (Approval No. WYYY-AEC-2022-050).

### Mice

C57BL/6J mice aged 6–8 weeks were obtained from Vital River (Beijing, China). T cell receptor transgenic mice specific for H–2IA^b^ OVA^323–339^ (OT-II) were purchased from Cyagen Biosciences (Santa Clara, CA, USA). *Srebf1*^fl/fl^ C57BL/6J mice (T009700) and *Cre*-*ERT2* mice (T050182) were generated by GemPharmatech (Nanjing, China) using CRISPR/Cas9. To create *Srebf1*^fl/fl^*Cre-ERT2* (cKO) mice, *Srebf1*^fl/fl^ mice were crossed with *Cre-ERT2* mice. These mice were bred to heterozygosity (*Srebf1*^fl/fl^*CreERT2*^*+/−*^) and underwent 10 rounds of backcrossing onto a B6 background to minimize unpredictable confounding factors. For the deletion of the floxed-Srebf1 gene, 100 mg/kg tamoxifen was injected intraperitoneally daily for seven consecutive days, followed by one week of housing before conducting subsequent experiments. For the animal experiments, 6- to 8-week-old Srebf1 cKO mice were compared with littermate controls that expressed wild-type (WT) SREBF1 (*Srebf1*^fl/fl^).

All mice were maintained under specific pathogen-free conditions in ventilated cages with controlled temperature and humidity (24 ± 2 ℃, 40–70% relative humidity) on a 12-hour light/dark cycle. C57BL/6J mice were allocated to the sham and CLP groups, randomly. WT and cKO mice were randomly assigned into four groups: WT, WT-CLP, cKO, and cKO-CLP. At the end of the experiment, mice were euthanized using carbon dioxide gas.

### CLP

To construct a septic model, we followed a previously outlined procedure for CLP [20]. Briefly, after anesthesia with 2.5% isoflurane inhalation, the abdominal cavity of mice was exposed, ligated 1.5 cm from the end of the cecum, and perforated with an 18-gauge needle. A small amount of fecal content was extruded before the ligature segment was placed back. After closing the abdominal cavity with 5–0 silk sutures, 5 mL/100 g saline was used to resuscitate the mice. The same process was followed in the sham-operated group, except that CLP was absent.

### Isolation of DCs and CD4^+^ T cells from murine spleens

Spleens were harvested from the mice, and splenic mononuclear cells were extracted using a Mouse Spleen Mononuclear Cell Isolation Kit (LDS1090PK, TBDscience, China). Mononuclear cells were incubated with CD11c (130-125-835, Miltenyi Biotech, Bergisch Gladbach, Germany) and CD4 microbeads (130-117-043, Miltenyi Biotech) to sort the splenic DCs and CD4^+^ T cells, respectively.

### BMDCs generation in vitro

BMDCs were generated as previously described [21]. Briefly, cells were rinsed out from the bone marrow of the femurs and tibias isolated from mice using RPMI-1640 medium (Gibco, Rockville, MD, US) and then resuspended in RPMI-1640 medium containing 10% fetal bovine serum (Gibco), recombinant mouse GM-CSF (20 ng/mL; AF-315-03-20, PeproTech, Rocky Hill, NJ, US), and murine IL-4 (1 ng/mL; AF-214-14-20, PeproTech). The medium was replenished twice during culture. Non-adherent cells were harvested on the seventh day and used in further experiments.

### Human peripheral blood mononuclear cells (PBMCs) isolation

Sepsis was diagnosed based on the Third International Consensus Definitions for Sepsis and Septic Shock (Sepsis-3) [1]. PBMCs were obtained from patients with sepsis and healthy controls at the First Affiliated Hospital of the Wenzhou Medical University. PBMCs were isolated using the human PBMC isolation solution (LTS1077, TBDsciences). The cells were then lysed with TRIzol reagent (15596026CN, Invitrogen, Carlsbad, CA, US) or seeded onto sterile coverslips pre-coated with poly-L-lysine for subsequent treatment.

### Transfection of small interfering RNA (siRNA)

SREBF1 siRNA (5’- CCCGCUGCUUUAAAGAUGUTT-3’) and negative control siRNA were purchased from GenePharma (Suzhou, China) to silence SREBF1 expression in BMDCs. Transient transfection of siSREBF1 into BMDCs was performed using RFect siRNA/miRNA Transfection Reagent (11026, Baidai Biotech, China) according to the manufacturer’s instructions. BMDCs were harvested 48 h after transfection for downstream analyses. Knockdown efficiency was confirmed by quantitative polymerase chain reaction (qPCR) and western blotting.

### Lipid droplet (LD) staining

LD staining was used to detect intracellular lipid content. For Oil Red O staining, DCs were stained using an Oil Red O Staining Kit (C0158S, Beyotime, China), according to the manufacturer’s instructions. For BODIPY staining, DCs were fixed with 4% paraformaldehyde and then incubated with BODIPY 493/503 (D3922, Invitrogen) for 30 min. After nuclear staining with DAPI, DCs were captured with the laser confocal scanning microscope (C2, Nikon, Tokyo, Japan).

### Immunoblots

Cells were collected, washed twice, and lysed in radioimmunoprecipitation assay (RIPA) buffer supplemented with protease and phosphatase inhibitors. After centrifugation of the cell lysates, a BCA assay kit (23227, Thermo Scientific, Waltham, MA, US) was used to quantify the proteins. Equal amounts of protein were separated using SDS-PAGE. Proteins were transferred onto polyvinylidene difluoride membranes (IPVH00010, Millipore, Billerica, MA, US) and blocked for 2 h at room temperature using 5% non-fat milk. Subsequently, the membranes were incubated overnight at 4 °C and treated with primary antibodies. The specific antibodies used were as follows: SREBF1 (1:1,000; sc-365514) was acquired from Santa Cruz Biotechnology (Dallas, TX, US). FASN (1:1,000; ab128870), ACACA (1:1,000; ab45174), SCD1 (1:1,000; ab236868), and Bcl-2 (1:2,000; ab182858) were purchased from Abcam (Cambridge, UK). Bax (1:1,000; 2772s), cleaved caspase-3 (1:1,000; 9661), PERK (1:1,000; 3192), p-eIF2α (1:1,000; 3398), eIF2α (1:1,000; 5324), and ATF4 (1:1,000; 11815) were from Cell Signaling Technology (Danvers, MA, US). GRP78 (1:2,000; 11587-1-AP), CHOP (1:1,000; 15204-1-AP), and β-Actin (1:5,000; 81115-1-RR) were purchased from ProteinTech (Chicago, IL, US). p-PERK (1:500; TA4499M) was purchased from Abmart (Shanghai, China). After rinsing, the membranes were incubated with the appropriate secondary antibodies for 1 h at room temperature. The iBright Imaging System (CL1500, Invitrogen) was used to visualize the protein bands, and ImageJ was used to perform the semi-quantification process. For each target protein, the expression level was normalized to β-actin from the same blot.

### Carboxyl fluorescein succinimidyl ester staining

CD4^+^ T cells were incubated in PBS containing 1 µM 5,6-carboxyfluorescein diacetate succinimidyl ester (CFSE) staining for 20 min at room temperature and protected from light. The staining was quenched by adding five volumes of complete culture medium. Cells were pelleted by centrifugation and resuspended in pre-warmed complete culture medium. CFSE-labeled CD4^+^ cells were evenly seeded into 96-well plates with pretreated DCs and co-cultured for 72 h. Next, the cells were harvested and assayed using flow cytometry.

### Flow cytometry assay

The phenotypes of DCs, proliferation of CFSE-labeled CD4^+^ T cells, and apoptosis levels were measured using flow cytometry. For phenotypic staining, the cells were collected and incubated with PE-conjugated anti-mouse CD40 (12-04-1082, eBioscience, San Diego, CA, US), PE-conjugated anti-mouse CD80 (12-0801-82, eBioscience), FITC-conjugated anti-mouse CD86 (11-0862-82, eBioscience), and APC-conjugated anti-mouse MHCII (17-5320-82, eBioscience) for 30 min at 4 °C. Samples were then analyzed using a BD FACS CantoII (San Jose, CA, US). The gating strategies used are shown in Supplementary Fig. 1.

### Biochemical analysis

Mouse TNF-α, IL-1β, IL-4, IL-6, IL-12, and IFN-γ ELISA kits were acquired from Jianglai (Shanghai, China). The supernatants of the cultured cells were collected and analyzed according to the manufacturer’s instructions. Free fatty acid (FFA), triglyceride (TG), and total cholesterol (TC) assay kits were purchased from Mlbio (Shanghai, China). The FFA, TG and TC contents of DCs were measured according to the manufacturer’s instructions.

### Immunofluorescence staining

After washing and fixing with 4% paraformaldehyde for 30 min, the DCs were permeabilized with 0.1% Triton X-100 and blocked for 1 h with 5% BSA. The cells were then incubated with rabbit anti-GRP78 antibody (1:100; 11587-1-AP, ProteinTech) at 4 °C overnight and with Alexa Fluor 488-conjugated anti-rabbit antibody (1:1,000; ab150077, Abcam) for 1 h at room temperature in the dark. Nuclei were stained with DAPI. Photomicrographs were obtained using a fluorescence microscope (Nikon, Tokyo, Japan).

### Terminal Deoxynucleotidyl transferase dUTP nick-end labelling (TUNEL) assay

Induction of apoptosis in DCs was identified using a TUNEL assay kit (E-CK-A321, Elabscience) in accordance with the manufacturer’s standard protocol. Images were taken using a fluorescence microscope (Nikon) after the nuclei were stained with DAPI. The apoptosis index was quantified as the ratio of TUNEL-positive cells using ImageJ software.

### Transmission electron microscopy (TEM)

Pretreated cells were fixed with 2.5% glutaraldehyde for 1 h at 4 °C. After drying with ethanol, the sliced fixed samples were embedded in epoxy resin. Ultrathin slices were obtained using an ultramicrotome and stained with lead citrate and uranyl acetate. An HT7800 transmission electron microscope (Hitachi, Tokyo, Japan) was used to view the cells. For quantitative analysis, the width of the ER lumen was measured using ImageJ.

### RNA sequencing

Splenic DCs were harvested from the mice in the CLP and sham groups. To extract total RNA, DCs were treated with TRIzol reagent (15596018CN; Invitrogen). RNA-seq was performed by LC-Bio Technology Co. Ltd. (Hangzhou, China). Differentially expressed genes (DEGs) based on|log_2_FC| ≥ 0.5 and *P*<0.05 were identified using DESeq2. Gene Ontology (GO) and Kyoto Encyclopedia of Genes and Genomes analyses were carried out using the R ClusterProfiler package (version 3.18.1). Linked lipid metabolic pathways were identified by gene set enrichment analysis.

### Statistical analysis

All statistical analyses were performed using SPSS (version 25.0). A minimum of three independent experiments were performed. Data are presented as mean ± standard deviation (SD). Normality was assessed with the Shapiro–Wilk test, and homogeneity of variances with Levene’s test. For comparisons between two groups, an unpaired two-tailed Student’s t-test was used for data with normal distribution and equal variances. When the data were normally distributed but variances were unequal, Welch’s t-test was applied. For non-normally distributed data, the Mann–Whitney U test was used. For comparisons among more than two groups, One-way ANOVA followed by Tukey’s post hoc test was applied for normally distributed data with equal variances​​, and the Kruskal–Wallis test with Dunn’s correction was used as a nonparametric alternative. *P* < 0.05 was considered statistically significant; non-significant differences were denoted as ns.

Experimental groups were randomly assigned, and data collection and analysis were performed in a blinded manner to minimize bias.

## Results

### Sepsis induces a lipid biosynthesis program and upregulates SREBF1 expression in DCs

**Fig. 1:**
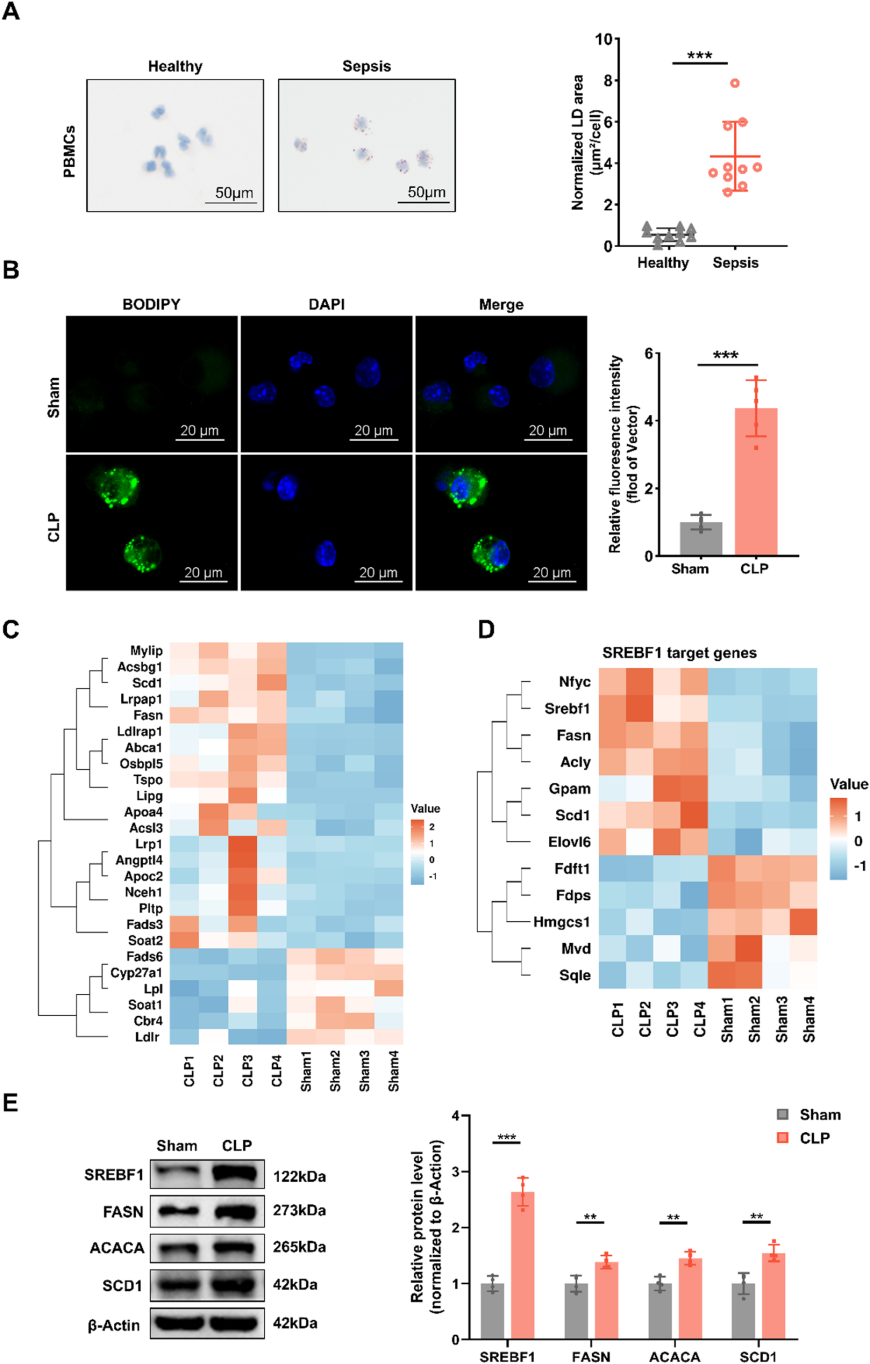
Sepsis induces lipid biosynthesis in DCs. (**A**) Representative Oil Red O staining images of LD in PBMCs from healthy controls and sepsis patients. Scale bars, 50 μm. Quantification of normalized LD area (µm²/cell) is shown (*n* = 10). (**B**) Representative fluorescence images of BODIPY 493/503-stained LDs in splenic DCs 24 h post-CLP or sham operation (*n* = 5). Scale bars, 20 μm. (**C**) Heatmap showing expression profiles of lipid biosynthesis–related DEGs in splenic DCs treated as in (**B**). (**D**) Heatmap showing expression of SREBF1 target genes in splenic DCs treated as in (**B**). (**E**) Immunoblot analysis of SREBF1, FASN, ACACA, and SCD1 protein levels in splenic DCs treated as in (**B**) (*n* = 4). Data are presented as the mean ± SD; *, *P* < 0.05; **, *P* < 0.01; ***, *P* < 0.001. PBMC, peripheral blood mononuclear cell; CLP, cecal ligation and puncture

To investigate alterations in lipid metabolism in septic DCs, we collected PBMCs from sepsis patients and healthy controls. Intracellular LD content was determined by Oil Red O staining. LD content was significantly increased in PBMCs from sepsis patients compared to healthy controls (Fig. 1A), indicating lipid metabolic reprogramming under septic conditions. We also examined changes in lipid metabolism in murine DCs. Similarly, LD accumulation was significantly increased in DCs following CLP (Fig. 1B), confirming lipid biosynthesis activation in septic DCs.

To identify key genes associated with lipid biosynthesis in DCs, splenic DCs were isolated from septic and sham-operated mice for RNA sequencing. Although lipid biosynthesis was not among the top 10 enriched GO terms, several lipid metabolism–related DEGs were significantly upregulated in septic DCs compared to the sham group (Fig. 1C). These findings are consistent with previous studies in other diseases [22] and suggest that lipid biosynthesis may be involved in regulating DC immune function during sepsis.

SREBF1 transcriptionally regulates lipogenic genes such as ACACA, FASN, and SCD1 [16, 23–25]. As shown in Fig. 1D, SREBF1 and its downstream target genes SCD1 and FASN were markedly upregulated. It is noteworthy that SREBF1 drives fatty acid production, a process essential for immune regulation [26]. However, the immunoregulatory role of SREBF1 in DCs remains unclear, prompting further investigation. To study the role of SREBF1, we first performed western blotting to assess its protein expression. As expected, SREBF1, FASN, and SCD1 protein levels were markedly increased in DCs following CLP (Fig. 1E). Although ACACA mRNA expression remained unchanged, its protein levels were significantly elevated, suggesting potential post-transcriptional regulation. Consistently, PBMCs from sepsis patients exhibited increased mRNA expression of SREBF1, FASN, ACACA, and SCD1 (Supplementary Fig. 2), supporting the clinical relevance of lipid biosynthesis activation. These findings collectively indicate that SREBF1 activation in septic DCs drives DNL, contributing to intracellular lipid overload.

### Knockout of SREBF1 dramatically alters lipid homeostasis in DCs during sepsis

**Fig. 2:**
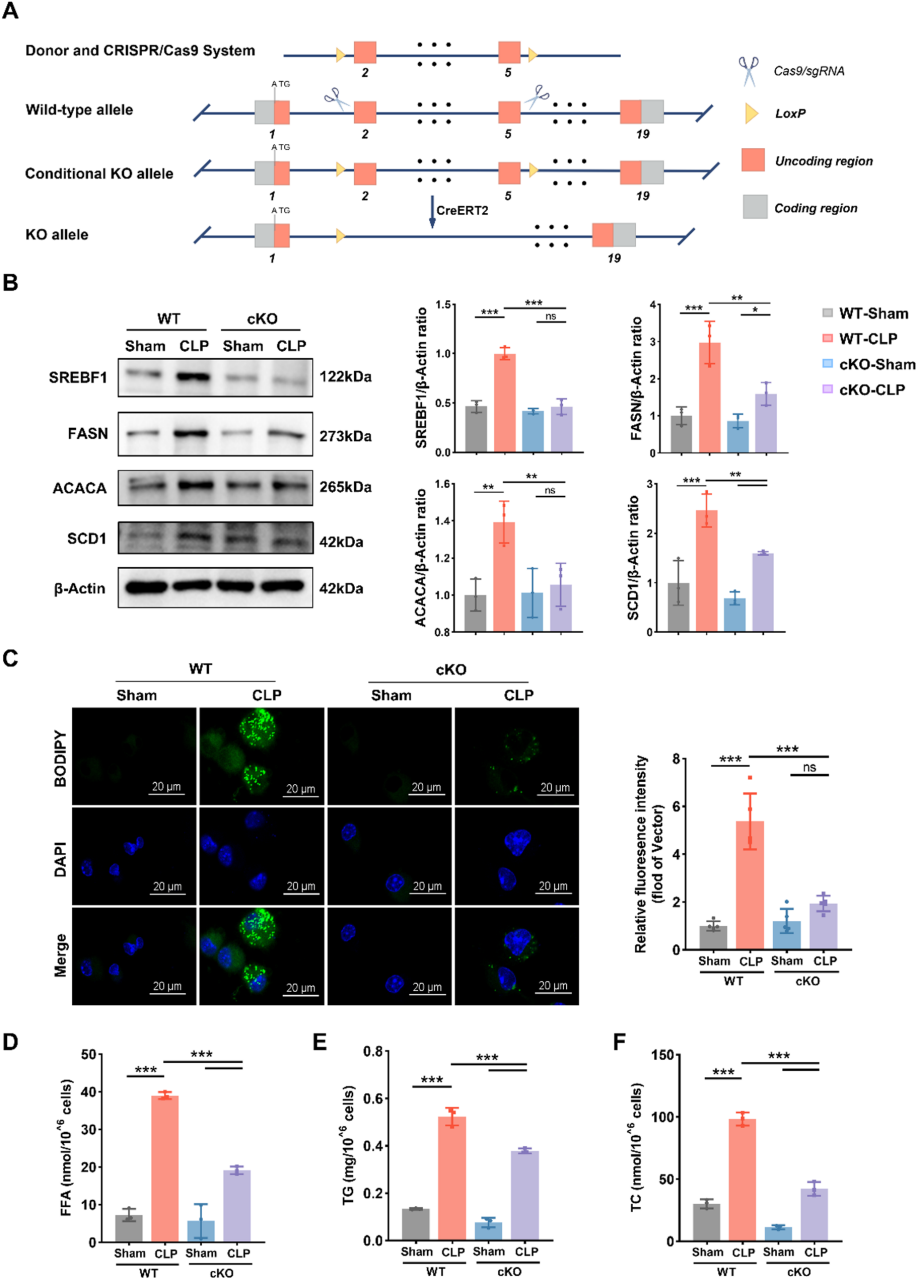
SREBF1 deletion reduces lipid biosynthesis in DCs after CLP. (**A**) Conditional knockout strategy for SREFB1 in cKO mice. (**B**) Immunoblot analysis of SREBF1, FASN, ACACA, and SCD1 protein levels in splenic DCs from WT and cKO mice 24 h after CLP or sham operation (*n* = 3). (**C**) Representative images of BODIPY 493/503-stained LDs in splenic DCs treated as in (**B**) (*n* = 5). Scale bars, 20 μm. (**D**) FFA llevels in splenic DCs treated as in (**B**) (*n* = 3). (**E**) TG levels in splenic DCs treated as (**B**) (*n* = 3). (**F**) TC levels in splenic DCs treated as in (**B**) (*n* = 3). Data are presented as the mean ± SD; ns, no significant difference; *, *P* < 0.05; **; *P* < 0.01; ***; *P* < 0.001. WT, wild type; cKO, conditional knockout; CLP, cecal ligation and puncture; FFA, free fatty acid; TG, triglyceride; TC, total cholesterol

SREBF1 regulates lipid metabolism by promoting DNL and cholesterol uptake [27]. We aimed to explore how SREBF1 regulates lipid metabolism in DCs during sepsis. A CLP-induced sepsis model was established using WT and tamoxifen-induced cKO mice (Fig. 2A). Splenic DCs were then isolated to assess the effect of SREBF1 deletion on lipid homeostasis. We first assessed the protein levels of SREBF1 and its downstream lipogenic enzymes ACACA, FASN, and SCD1 in splenic DCs from WT and cKO mice using western blotting (Fig. 2B). SREBF1 expression was markedly decreased in cKO DCs, confirming effective gene knockout. The expression of ACACA, FASN, and SCD1 was also reduced, consistent with the regulatory role of SREBF1 in lipogenesis. As expected, BODIPY staining showed a marked reduction in both the size and number of LDs in cKO DCs (Fig. 2C). We further quantified intracellular levels of FFA, TG, and TC in DCs (Fig. 2D, E). These findings show that SREBF1 deletion disrupted lipid homeostasis in septic DCs, as reflected by decreased fatty acid and cholesterol levels.

### SREBF1 negatively regulates the immune function of DCs in septic mice

**Fig. 3:**
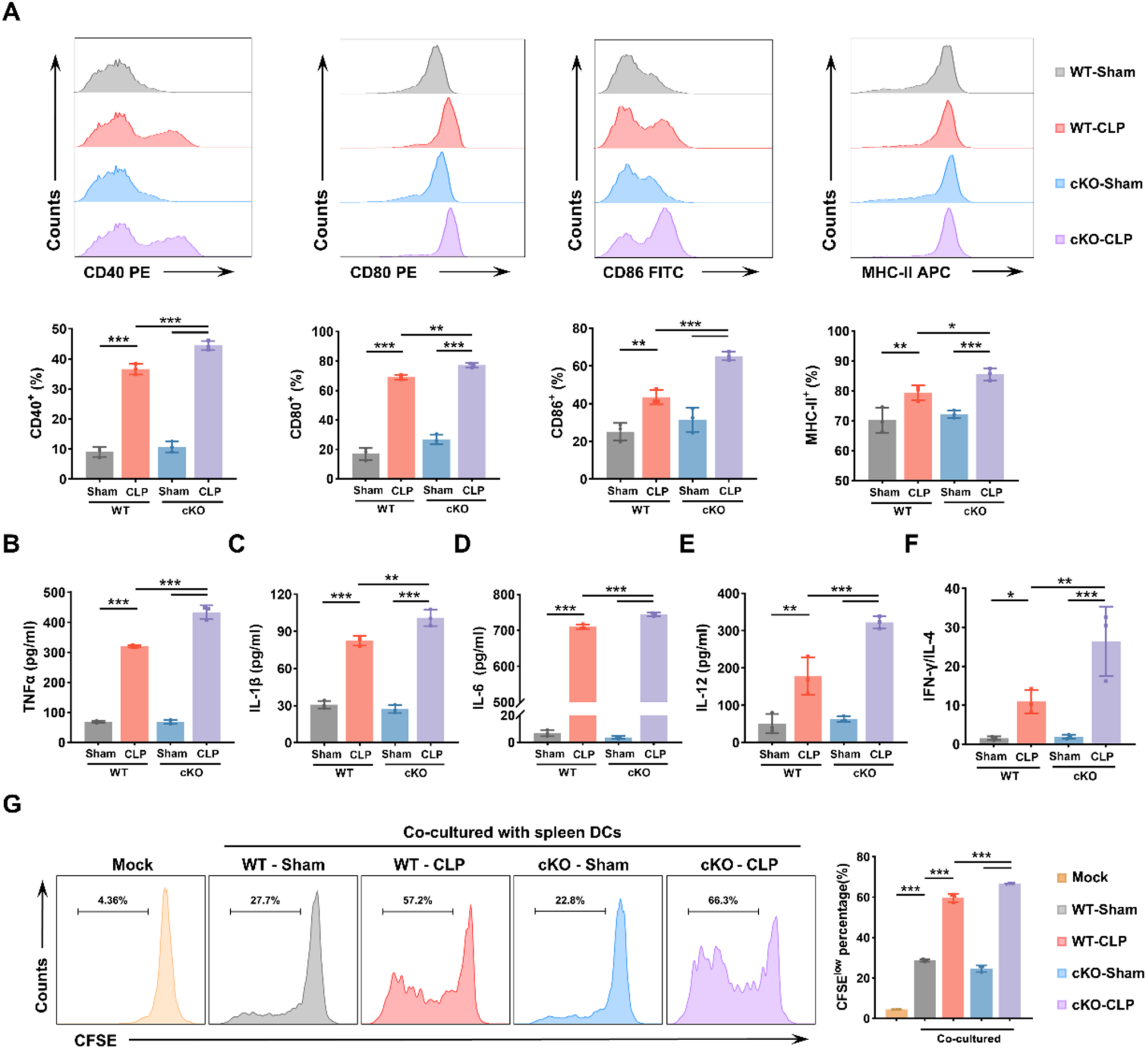
SREBF1 knockout restores DC immune function following CLP. (**A**) Flow cytometric analysis of co-stimulatory molecule expression (CD40, CD80, CD86, and MHC II) in splenic DCs from WT and cKO mice 24 h after CLP or sham operation (*n* = 3). (B–E) Splenic DCs were treated as described in (**A**) and cultured for 6 h. Cytokine levels (TNF-α, IL-1β, IL-6, and IL-12)in the supernatants were measured by ELISA (*n* = 3). (**F**) CD4^+^ T cells were co-cultured with DCs (DC: T cell ratio = 1:100) treated as in (**A**). IFN-γ and IL-4 levels in the supernatant were quantified by ELISA, and the IFN-γ/IL-4 ratio was calculated to evaluate Th1/Th2 polarization (*n* = 3). (**G**) CFSE labeling and flow cytometric analysis of CD4^+^ T cell proliferation induced by splenic DCs treated as in (**A**) (*n* = 3). Data are presented as the mean ± SD; *, *P* < 0.05; **, *P* < 0.01; ***, *P* < 0.001. WT, wild type; cKO, conditional knockout; CLP, cecal ligation and puncture; DC, dendritic cell; CFSE, 5,6-carboxyfluorescein diacetate succinimidyl ester

To investigate the role of SREBF1 in septic DCs, we evaluated phenotypic maturation, cytokine production, and CD4^+^ T cell activation in splenic DCs isolated from WT and cKO mice post-CLP or sham surgery. Flow cytometry revealed that the cKO-CLP group had a significantly higher proportion of DCs expressing CD40, CD80, CD86, and MHC II than WT-CLP controls (Fig. 3A; gating strategies as detailed in Supplemental Fig. 1). Production of TNFα, IL-1β, IL-6, and IL-12 in DCs was also increased by SREBF1 knockout (Fig. 3B-E). We further co-cultured the harvested DCs with CD4^+^ T cells. CD4^+^ T cells from the cKO-CLP group exhibited a marked increase in the IFN-γ/IL-4 ratio compared to WT-CLP controls (Fig. 3F), indicating a shift toward T helper 1 (Th1) polarization. Consistent with this, CFSE analysis demonstrated that SREBF1 knockout enhanced the proliferative capacity of CD4⁺ T cells after CLP (Fig. 3G).

These findings were further validated in vitro by siRNA-mediated knockdown of SREBF1 in BMDCs, followed by LPS stimulation. Consistent with the in vivo results, SREBF1 silencing significantly upregulated the surface expression of CD40, CD80, CD86, and MHC II, and increased secretion of TNF-α, IL-1β, IL-6, and IL-12 (Supplementary Fig. 4A–E). Furthermore, SREBF1-silenced BMDCs more effectively promoted CD4⁺ T cell proliferation and Th1 polarization, as reflected by an increased IFN-γ/IL-4 ratio and CFSE labeling (Supplementary Fig. 4F–G). Together, these results from both in vivo and in vitro models demonstrate that SREBF1 promotes immunoparalysis of DCs in sepsis.

### SREBF1 promotes DC apoptosis in septic mice

**Fig. 4:**
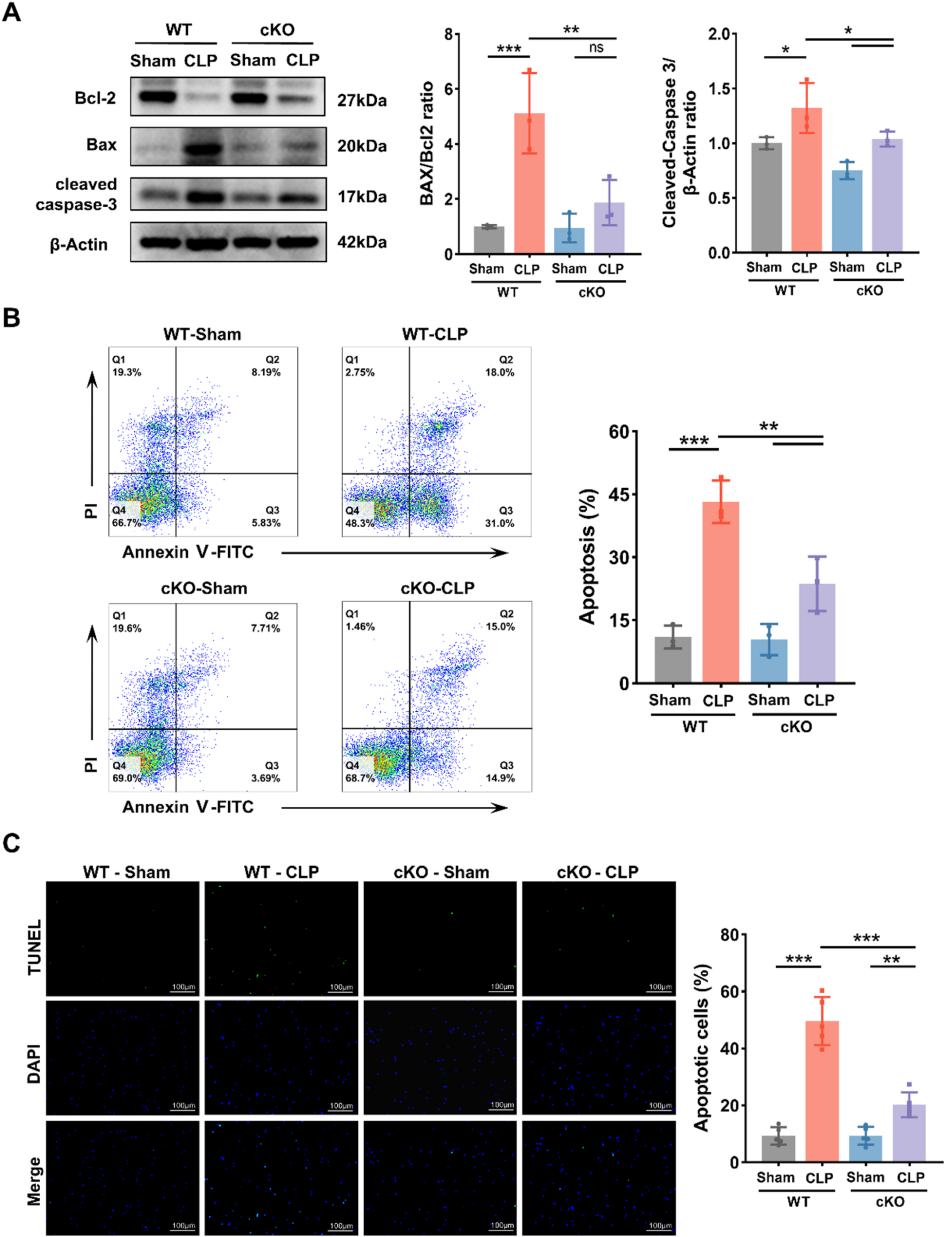
SREBF1 knockout attenuates apoptosis in splenic DCs after CLP. (**A**) Expression levels of Bcl-2, Bax, and Cleaved caspase-3 were analyzed by immunoblot in splenic DCs from WT and cKO mice 24 h after CLP or sham operation (*n* = 3). (**B**) Splenic DCs were treated as described in (**A**), stained with Annexin V-FITC/PI, and analyzed for apoptosis by flow cytometry (*n* = 3). (**C**) Representative images of TUNEL-stained splenic DCs treated as in (**A**) (*n* = 5). Scale bars, 100 μm. Data are presented as the mean ± SD; ns, no significant difference; *, *P* < 0.05; **, *P* < 0.01; ***, *P* < 0.001. WT, wild type; cKO, conditional knockout; CLP, cecal ligation and puncture

In addition to exhibiting impaired immune function, septic DCs showed a marked reduction in cell number [28, 29]. DC apoptosis has been shown to contribute to immune dysfunction and organ injury during sepsis [30]. However, whether SREBF1 contributes to DC apoptosis during sepsis remains unknown. To evaluate the role of SREBF1 in DC apoptosis during sepsis, we examined the expression of apoptosis-related proteins in DCs from WT and cKO mice. Western blot analysis revealed reduced cleaved caspase-3 expression and a lower Bax/Bcl-2 ratio in DCs from the cKO-CLP group compared to the WT-CLP group (Fig. 4A), indicating that SREBF1 knockout alleviates apoptosis in septic DCs. Flow cytometry further confirmed a significantly lower apoptotic rate in the cKO-CLP group, as assessed by Annexin V-FITC/PI staining (Fig. 4B). This finding was corroborated by TUNEL assay results (Fig. 4C). In line with the in vivo results, siRNA-mediated SREBF1 knockdown attenuated LPS-induced apoptosis in DCs in vitro (Supplementary Fig. 5). These findings suggest that SREBF1 promotes DC apoptosis, contributing to the loss of DCs and subsequent immunosuppression in sepsis.

### SREBF1 modulates ER stress through a lipid-dependent pathway in DCs

**Fig. 5:**
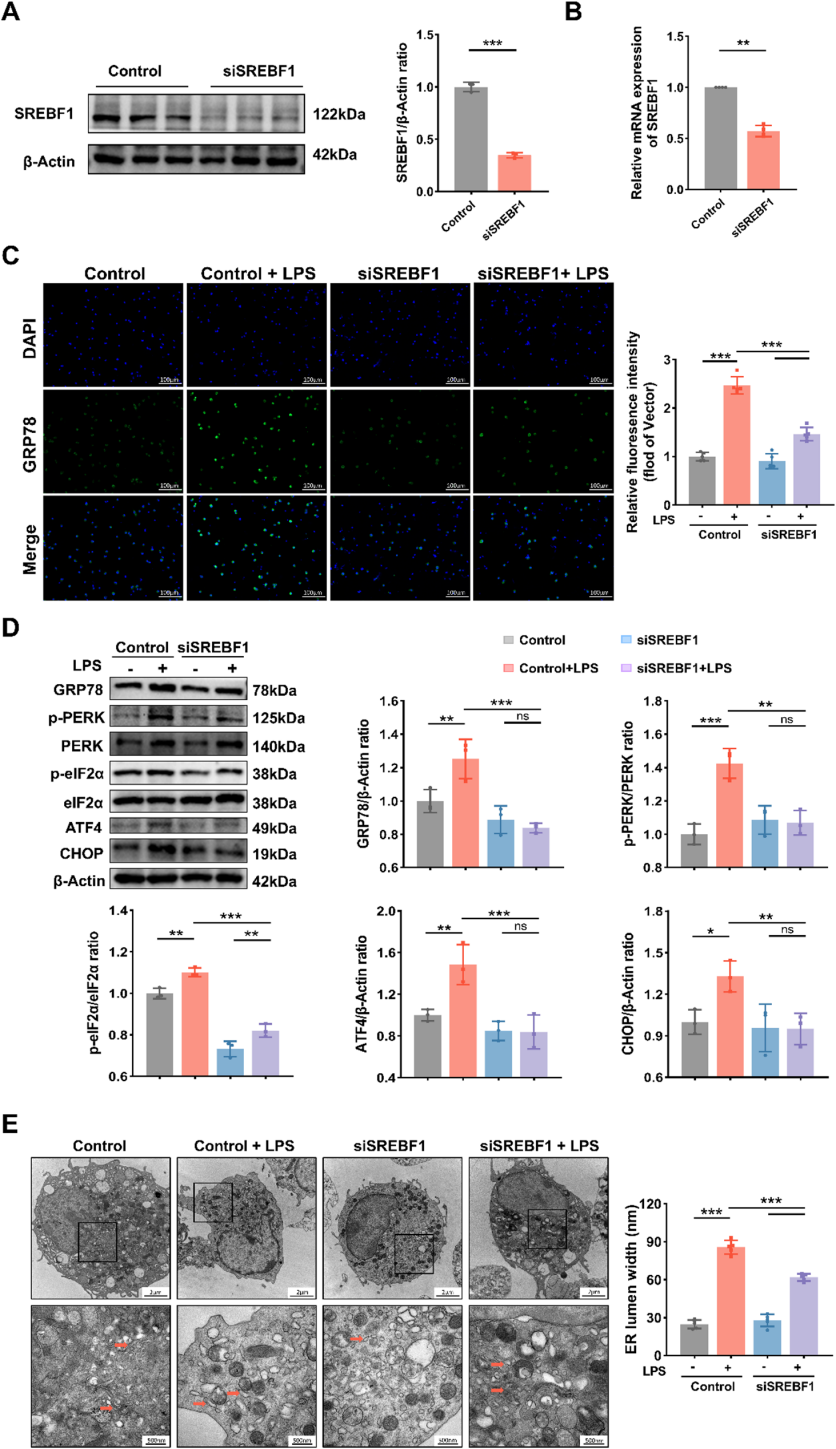
SREBF1 activates ER stress in BMDCs through the PERK/eIF2α/ATF4/CHOP pathway. (**A**) Western blot analysis of SREBF1 expression in control and siSREBF1-treated BMDCs (*n* = 3). (**B**) qPCR analysis of SREBF1 mRNA expression in BMDCs as described in (**A**) (*n* = 4). (**C**) BMDCs were transfected with negative control siRNA or siSREBF1 and stimulated with 100 ng/mL LPS for 12 h. Representative fluorescence images showing GRP78 expression. Scale bar, 100 μm. Relative fluorescence intensity was quantified (*n* = 5). (**D**) Western blot analysis of ER stress–related proteins (GRP78, p-PERK, PERK, p-eIF2α, eIF2α, ATF4, and CHOP) in BMDCs treated as described in (**C**) (*n* = 3). (**E**) Representative TEM images of ER morphology in BMDCs treated as in (**C**). Scale bars, 2 μm and 500 nm. ER lumen width was quantified (*n* = 5). Data are presented as the mean ± SD; *, *P* < 0.05; **, *P* < 0.01; ***, *P* < 0.001. LPS, lipopolysaccharide; ER, endoplasmic reticulum

LDs are physiologically and functionally linked to the ER, and lipid storage is essential for maintaining ER homeostasis. Disruption of lipid homeostasis inevitably triggers ER stress and activates the unfolded protein response (UPR), ultimately leading to cell death [31]. As shown in Fig. 5A–B, we first evaluated the efficiency of SREBF1 silencing by siRNA using western blotting and qPCR. To evaluate the effect of SREBF1 on ER stress activation, the expression of the ER chaperone GRP78 was assessed by immunofluorescence. Compared to the control + LPS group, GRP78 expression was markedly reduced in the siSREBF1 + LPS group (Fig. 5C). Specifically, the PERK/eIF2α/ATF4/CHOP pathway plays a crucial role in the transcriptional regulation of the ER stress response. We therefore investigated whether SREBF1 modulates this pathway in DCs. As shown in Fig. 5D, LPS stimulation upregulated GRP78, p-PERK, p-eIF2α, ATF4, and CHOP in the control group, whereas the expression levels of these proteins were markedly reduced in siSREBF1-treated cells. TEM analysis revealed pronounced ER expansion and vesiculation in the control group after LPS, which was alleviated by SREBF1 silencing (Fig. 5E). Quantitative analysis also confirmed a reduction in ER lumen width. These findings indicate that ER stress is activated in DCs during sepsis and can be alleviated by SREBF1 silencing through inhibition of the PERK/eIF2α/ATF4/CHOP pathway.

To assess whether SREBF1 broadly regulates ER stress or primarily affects lipid-associated pathways, DCs were treated with tunicamycin (TM), a classical inducer of ER stress. TM inhibits N-linked glycosylation in the ER by blocking the transfer of GlcNAc to dolichol phosphate, thereby impairing protein folding and triggering ER stress [32]. TM treatment markedly increased the expression of ER stress markers—including GRP78, p-PERK, p-eIF2α, ATF4, and CHOP—in both control and SREBF1-silenced DCs (Supplementary Fig. 6). Importantly, SREBF1 silencing failed to attenuate TM-induced ER stress, as the expression levels of these markers remained elevated after TM stimulation. This contrasts sharply with the suppression of LPS-induced ER stress by SREBF1 silencing, demonstrating that SREBF1 selectively regulates lipid-associated ER stress but not glycosylation inhibition-driven UPR.

### SREBF1 impairs the immune function of DCs by promoting lipid-associated ER stress

**Fig. 6:**
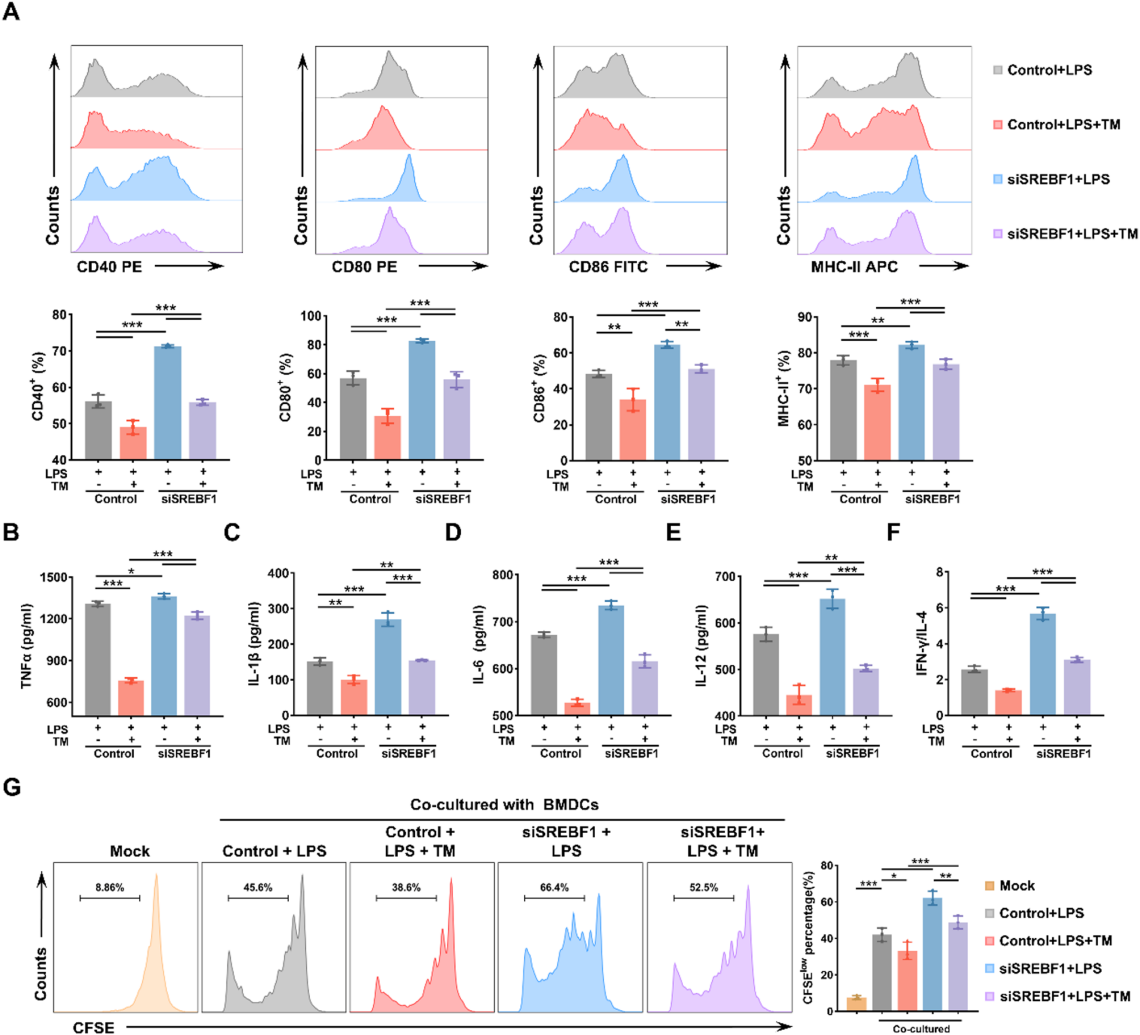
SREBF1 silencing alleviates ER stress-induced immunosuppression in BMDCs. (**A**) BMDCs were pretreated with or without tunicamycin (TM, 0.5 µg/mL) for 1 h, followed by stimulation with LPS (100 ng/mL) for 12 h. Co-stimulatory molecule expression (CD40, CD80, CD86, and MHC II) was analyzed by flow cytometry in control and siSREBF1-treated BMDCs (*n* = 3). (**B**–**E**) ELISA was used to quantify the levels of TNF-α, IL-1β, IL-6, and IL-12 in the supernatant of BMDCs treated as in (**A**) (*n* = 3). (**F**) CD4⁺ T cells were co-cultured with BMDCs (DC: T cell ratio = 1:100) treated as in (**A**). IFN-γ and IL-4 levels were measured by ELISA, and the IFN-γ/IL-4 ratio was calculated to evaluate Th1/Th2 polarization (*n* = 3). (**G**) CFSE labeling and flow cytometric analysis of CD4⁺ T cell proliferation induced by BMDCs treated as in (**A**) (*n* = 3). Data are presented as the mean ± SD; *, *P* < 0.05; **, *P* < 0.01; ***, *P* < 0.001. LPS, lipopolysaccharide; TM, tunicamycin; BMDC, bone marrow-derived dendritic cell; CFSE, 5,6-carboxyfluorescein diacetate succinimidyl ester

To further explore the role of the PERK/eIF2α/ATF4/CHOP pathway in SREBF1-mediated immunoparalysis, we examined the impact of TM-induced ER stress on DC function. As shown in Fig. 6A, LPS + TM treatment markedly reduced the expression of co-stimulatory molecules (CD40, CD80, CD86) and MHC II compared to LPS alone. Notably, SREBF1 silencing partially restored the expression of these markers in the siSREBF1 + LPS + TM group compared to the control + LPS + TM group. Consistently, ELISA revealed that TM significantly suppressed the secretion of inflammatory cytokines (TNF-α, IL-1β, IL-6, and IL-12) in LPS-stimulated DCs (Fig. 6B–E), whereas SREBF1 knockdown partially alleviated this suppression. Moreover, TM further impaired DC-mediated CD4⁺ T cell proliferation and Th1 polarization, as indicated by reduced T cell expansion and a lower IFN-γ/IL-4 ratio (Fig. 6F–G). Silencing of SREBF1 partially rescued these defects. Collectively, these results demonstrate that SREBF1 aggravates the immunoparalysis of DCs by enhancing ER stress, likely through the PERK/eIF2α/ATF4/CHOP pathway.

### SREBF1 Silencing mitigates DC apoptosis by alleviating lipid-induced ER stress following LPS stimulation

**Fig. 7:**
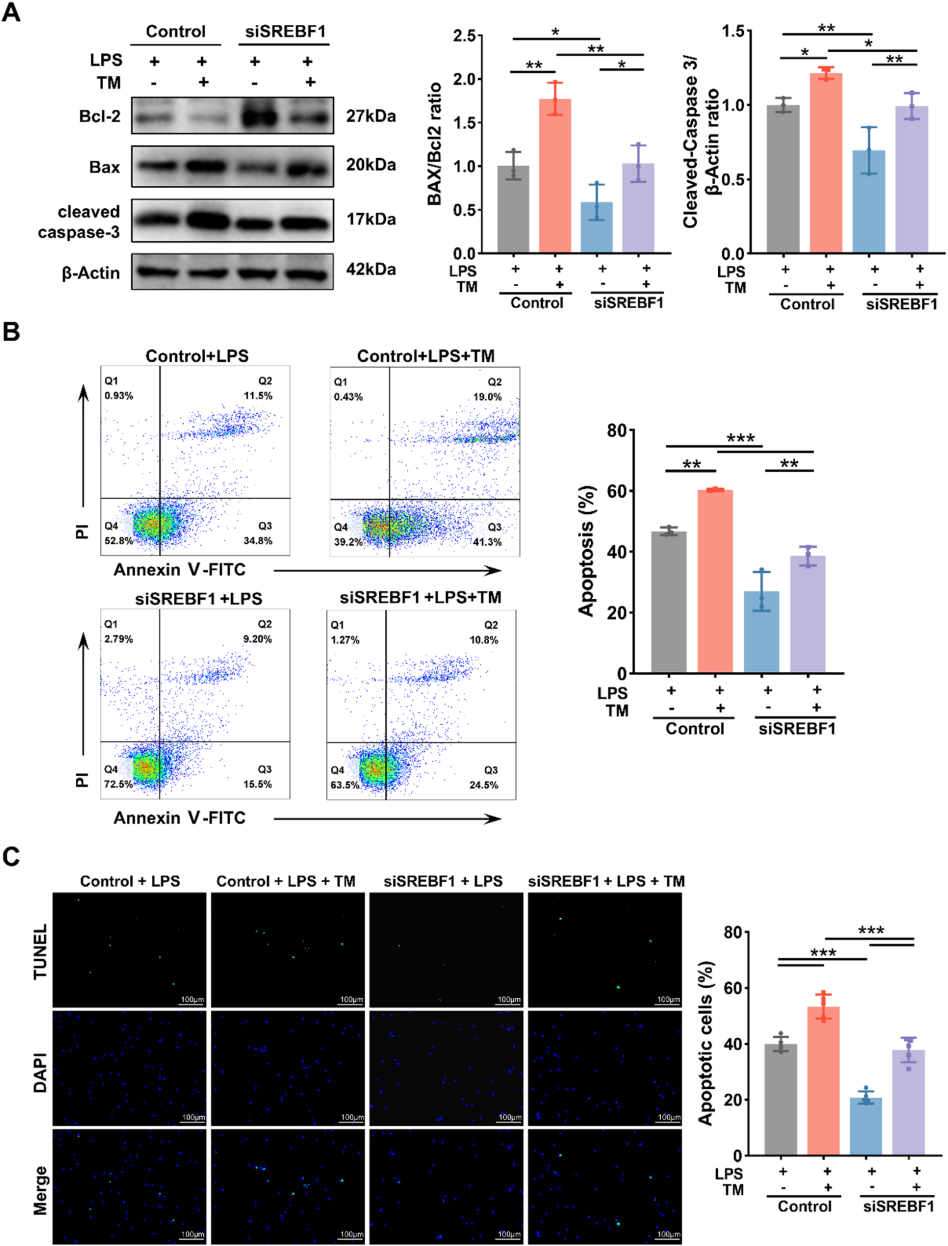
SREBF1 silencing reduces apoptosis of BMDCs by alleviating ER stress. (**A**) Immunoblot analysis of Bcl-2, BAX, and Cleaved caspase-3 protein expression in control and siSREBF1-treated BMDCs with or without TM (0.5 µg/mL) pretreatment for 1 h prior to LPS (100 ng/mL) stimulation for 12 h (*n* = 3). (**B**) Apoptosis in BMDCs treated as described in (**A**) were measured by flow cytometry (*n* = 3). (**C**) Representative TUNEL-stained images of BMDCs treated as in (**A**). Quantification of apoptotic cell percentages is show (*n* = 5). Scale bars, 100 μm. Data are presented as the mean ± SD; *, *P* < 0.05; **, *P* < 0.01; ***, *P* < 0.001. LPS, lipopolysaccharide; TM, tunicamycin

Given that activation of the PERK/eIF2α/ATF4/CHOP pathway is closely associated with apoptosis [33], we investigated whether SREBF1 silencing mitigates DC apoptosis by inhibiting the PERK pathway. As shown in Fig. 7A, the siSREBF1 + LPS + TM group exhibited a reduced Bax/Bcl-2 ratio and lower levels of cleaved caspase-3 compared to the control + LPS + TM group. A significant reduction in apoptosis was further confirmed by flow cytometry and TUNEL staining (Fig. 7B–C), supporting the role of SREBF1 in promoting apoptosis through ER stress pathways.

**Fig. 8:**
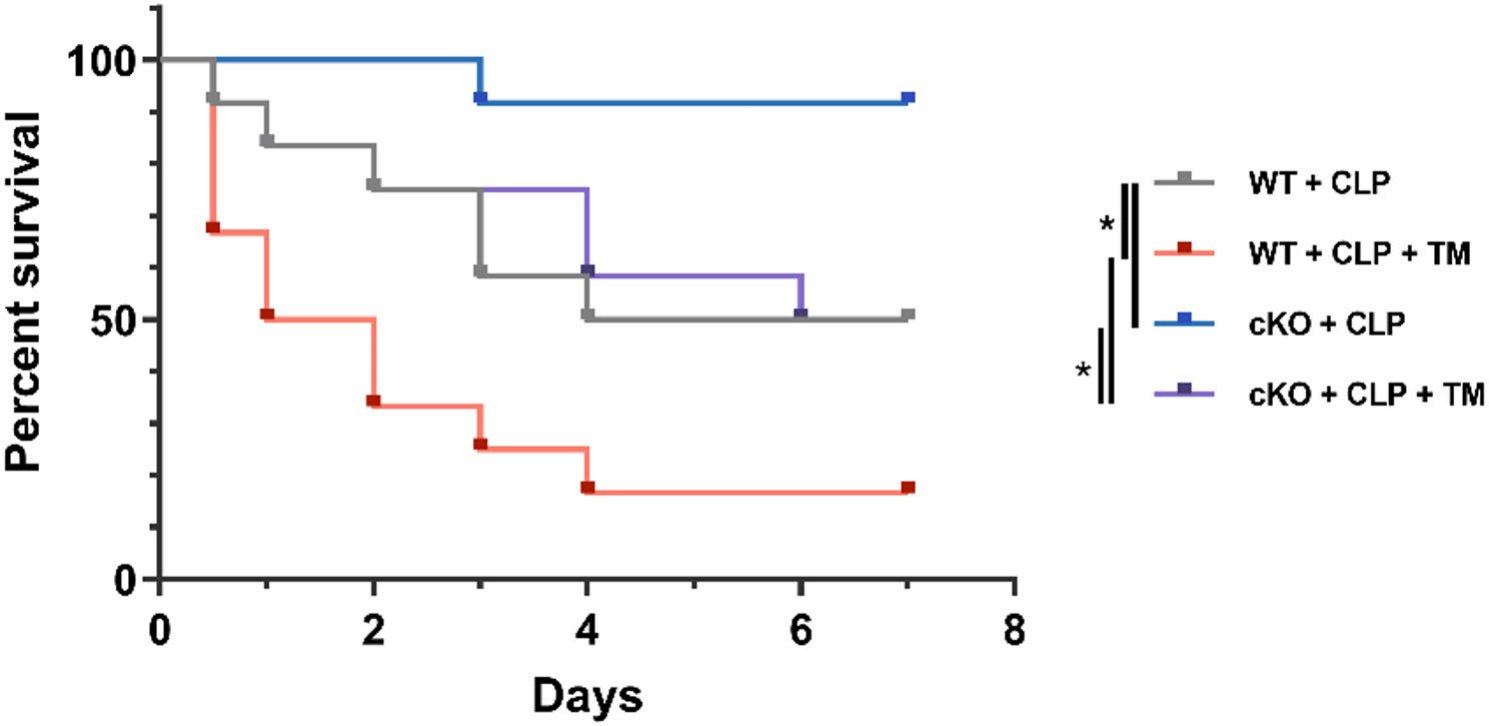
SREBF1 deficiency improves survival in CLP-induced sepsis by attenuating lipid-associated ER stress. WT and cKO mice were subjected to CLP with or without pretreatment with TM (1 mg/kg) 1 h before CLP (*n* = 12). Survival curves were analyzed by Kaplan-Meier method and compared using log-rank (Mantel-Cox) test. *, *P* < 0.05

To assess the physiological relevance of these findings, we examined survival outcomes in WT and SREBF1 cKO mice subjected to CLP with or without TM treatment. As shown in Fig. 8, cKO mice exhibited improved survival after CLP compared to WT controls. However, this benefit was only partially maintained in the CLP + TM group. This partial rescue aligns with our mechanistic findings that CLP-induced mortality involves SREBF1-driven lipid overload and activation of the PERK/eIF2α/ATF4/CHOP pathway (Fig. 5), which is reversed in cKO mice. In contrast, TM induces ER stress through glycosylation blockade, independently of SREBF1, as shown by comparable ER stress marker expression in both WT and SREBF1-silenced DCs (Supplementary Fig. 6).

Collectively, these results demonstrate that SREBF1 promotes DC apoptosis and sepsis-related mortality by exacerbating lipid-induced ER stress but does not broadly suppress ER stress from unrelated triggers like TM.

## Discussion

Our study reveals that SREBF1-mediated lipid metabolism reprogramming drives DC dysfunction in sepsis via ER stress. While previous research has linked lipid metabolism to DC activity, our work uniquely identifies SREBF1 as the central transcriptional regulator of this process in the context of sepsis. This work provides new insights into how lipid biosynthesis and ER stress pathways intersect to influence DC immunoparalysis in sepsis, and opens up novel avenues for therapeutic interventions.

Immunosuppression contributes to the pathogenesis of sepsis, with DCs playing a key role in this process [34–37]. Thus, targeting DCs to regulate the host immune response following septic insult is of significant therapeutic interest. Emerging evidence highlights the complex role of lipid metabolism reprogramming in the regulation of DC function. DCs with elevated lipid content exhibit impaired antigen processing, which can severely hinder their immune functions. Treating DCs with an ACACA inhibitor to reduce lipid levels restores their functionality and significantly boosts T cell immune response capabilities [14]. Contrasting evidence suggests that DC activation requires the DNL, a process essential for the acquisition of an immunogenic phenotype [38]. These findings highlight the need to better understand how lipid metabolism regulates DCs, particularly in the context of sepsis-induced immunosuppression. In this study, we investigated lipid biosynthesis reprogramming in DCs during sepsis. Initially, we observed a remarkable increase in both the quantity and size of LDs in human PBMCs and mouse splenic DCs during sepsis. RNA sequencing confirmed these observations, revealing elevated mRNA levels of SREBF1 and its downstream enzymes (FASN and SCD1), which are key regulators of fatty acid metabolism. To explore the role and mechanisms of SREBF1-mediated lipid biosynthesis in DCs, we generated Srebf1 cKO mice using CRISPR/Cas9 technology. We found that SREBF1 deficiency reduced both lipid content and the expression of lipid biosynthesis enzymes in DCs. These results demonstrate that SREBF1 plays a crucial role in regulating fatty acid synthesis in DCs, particularly during sepsis.

SREBF1 not only regulates lipid homeostasis but also plays a potential role in immune regulation. SREBF1 promotes lipogenesis in macrophages in response to LPS and directly activates the gene encoding Nlrp1a, a key inflammasome component whose absence impairs inflammasome function [18]. Moreover, the canonical T helper 2 cell cytokine IL-4 activates SREBF1, which enhances DNL in macrophages as part of alternative activation [19]. SREBPs are essential for coordinating T-cell receptor signaling with lipid metabolism to support T cell growth and proliferation during activation [39]. While SREBF1 has been implicated in lipid metabolism and immune regulation in macrophages and T cells, its role in DCs remains poorly understood. In this work, SREBF1 knockout alleviated the immune dysfunction and apoptosis of DCs in sepsis. SREBF1-deficient DCs showed increased expression of co-stimulatory molecules (CD40, CD80, CD86, and MHC II) and enhanced secretion of pro-inflammatory cytokines (TNFα, IL-1β, IL-6, and IL-12) following CLP. DCs, as key APCs, bridge innate and adaptive immunity. Thus, SREBF1 knockout markedly enhanced the capacity of DCs to promote CD4^+^ T cell proliferation. SREBF1-deficient DCs also exhibited reduced apoptosis during sepsis. Our data indicate that SREBF1 is involved in DC immunoparalysis, thus exacerbating sepsis severity.

The ER is essential for regulating cellular ion homeostasis, protein synthesis, quality control, and protective signal transduction, which are crucial for cell survival and the initiation of programmed cell death [40]. ER stress is triggered by disruptions in cellular homeostasis, including the accumulation of immature or misfolded proteins, Ca^2+^ overload, and nutrient deprivation [41]. It has been reported that lipid overload disrupts antigen presentation in DCs by inducing ER stress [14]. For example, Cubillos-Ruiz et al. have demonstrated that ER stress-induced Xbp1 splicing is associated with lipid build-up in DCs, leading to defective DC antigen presentation [42]. Additionally, an increase in lipid concentration may contribute significantly to ER stress and cell damage by promoting Ca^2+^ loss from the ER [43]. Consistent with previous studies [44], we observed elevated ER stress in septic DCs, accompanied by increased lipid biosynthesis. Mechanistically, SREBF1-mediated lipid overload activated the PERK/eIF2α/ATF4/CHOP pathway, a canonical ER stress axis, thereby promoting DC dysfunction and apoptosis. To directly assess the role of ER stress in this process, we used TM, a classical ER stress inducer that blocks N-linked glycosylation [32]. TM partially reversed the effects of SREBF1 silencing, directly supporting the causal relationship between SREBF1-mediated lipid biosynthesis and PERK pathway activation. This rescue experiment strengthened our conclusion that SREBF1 mediates its effects primarily through ER stress modulation. However, our study showed that SREBF1 silencing abolished LPS-induced ER stress but failed to attenuate TM-induced ER stress. This divergence reveals that SREBF1 selectively regulates lipid-associated ER stress, but not glycosylation blockade–driven ER stress. This specificity was further supported by survival data in CLP models. While SREBF1 knockout improved survival in septic mice, the benefit was only partial when TM was co-administered, reflecting the inability of SREBF1 deletion to counteract glycosylation-dependent ER stress. Together, these findings demonstrate that SREBF1 acts upstream to initiate lipid-driven ER stress and identify it as a selective regulator of metabolic ER stress pathways in sepsis.

There are several limitations that warrant further exploration. First, the systemic knockout of SREBF1 used in this study limits our ability to pinpoint the exact contribution of SREBF1 in DCs versus other immune cells or organs. Future studies using DC-specific SREBF1 knockout models could help clarify its role more precisely. Nevertheless, SREBF1 may also influence the function of other immune cells beyond DCs. The results obtained by systemic knockout still have a certain degree of generalizability and can provide important clues to understand the central role of SREBF1 in immune regulation. Second, our study relied on the CLP model, which primarily mimics polymicrobial sepsis. Future studies should validate these findings in other sepsis models (e.g., LPS-induced endotoxemia) to assess generalizability. Third, the human PBMC data, while suggestive, require confirmation in patient-derived DCs to establish clinical relevance. Finally, whether SREBF1-induced lipid accumulation directly disrupts ER membrane integrity or alters calcium homeostasis, thereby initiating ER stress, requires further investigation.

## Conclusion

Our study identifies SREBF1 as a master regulator of DC dysfunction in sepsis by inducing lipid biosynthesis-driven ER stress via the PERK/eIF2α/ATF4/CHOP axis. These findings not only advance our understanding of immunometabolic crosstalk in sepsis but also identify SREBF1 as a promising therapeutic target. Pharmacological inhibition of SREBF1 (e.g., fatostatin) or its downstream ER stress effectors (e.g., PERK, eIF2α, ATF4, and CHOP) may offer a novel strategy to restore immune competence and improve survival in septic patients.

## Electronic supplementary material

Below is the link to the electronic supplementary material.

Supplementary Material 1

## Data Availability

No datasets were generated or analysed during the current study.
