# Supplementary material for: SREBF1 mediates immunoparalysis of dendritic cells in sepsis by regulating lipid metabolism and endoplasmic reticulum stress
Source: Cell Commun Signal. 2025 Jun 16;23:286. doi: 10.1186/s12964-025-02295-9 (PMC12172212; doi:10.1186/s12964-025-02295-9)
Supplement: Supplementary file 1 — Supplementary Material 1 [file 12964_2025_2295_MOESM1_ESM.docx]

**SREBF1 mediates immunoparalysis of dendritic cells in sepsis by regulating lipid metabolism and endoplasmic reticulum stress**

Yaolu Zhang ^1, a, b^, Fangfang Wu ^1, a, b^, Yan Li ^a, b^, Jiaxin Liu ^a, b^, Liuyan Zhu ^a, b^, Min Zhang ^a, b^, Zhongqiu Lu ^a, b, *^

^a^ Emergency Department, the First Affiliated Hospital of Wenzhou Medical University, Wenzhou, 325000, China

^b^ Wenzhou Key Laboratory of Emergency and Disaster Medicine, Wenzhou, 325000, China

^*^ Corresponding author

Emergency Department, the First Affiliated Hospital of Wenzhou Medical University, Ouhai District, Wenzhou, 325000, China.

E-mail address: lzq_640815@163.com

^1^ Yaolu Zhang and Fangfang Wu contributed equally to this work.


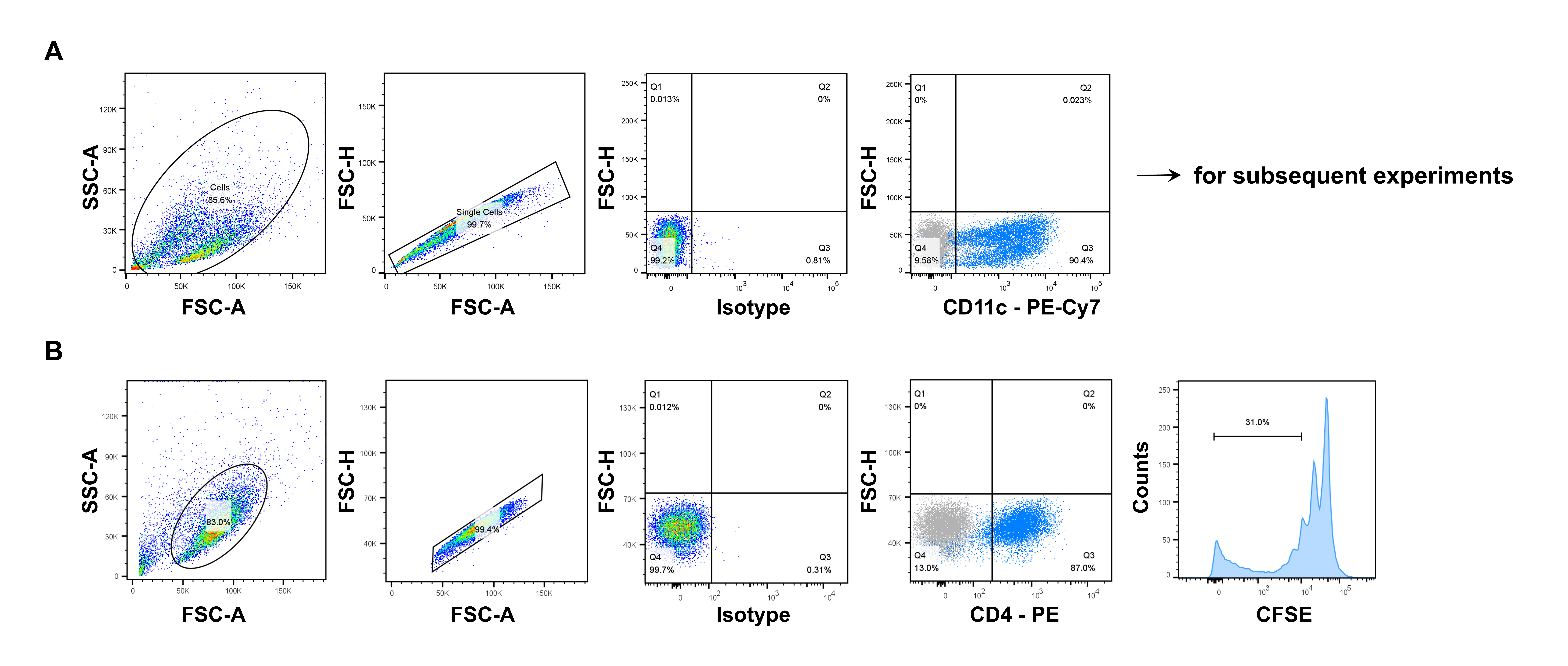


**Supplementary Figure 1.** Gating strategies for flow cytometry analysis.
(A) DC gating strategy. Cells were first gated based on FSC-A vs. SSC-A to exclude debris, followed by FSC-A vs. FSC-H to eliminate doublets. CD11c⁺ DCs were identified ​​by assessing PE-Cy7 fluorescence intensity relative to isotype-matched controls. The CD11c⁺ population was used for subsequent analysis of surface markers expression and apoptosis. (B) CD4⁺ T cell gating strategy. Lymphocytes were selected using FSC-A vs. SSC-A, followed by FSC-A vs. FSC-H to remove doublets. CD4⁺ T cells were identified by comparison with isotype controls for CD4-PE fluorescence. CFSE dilution within the CD4⁺ T cell population was then analyzed to assess T cell proliferation.


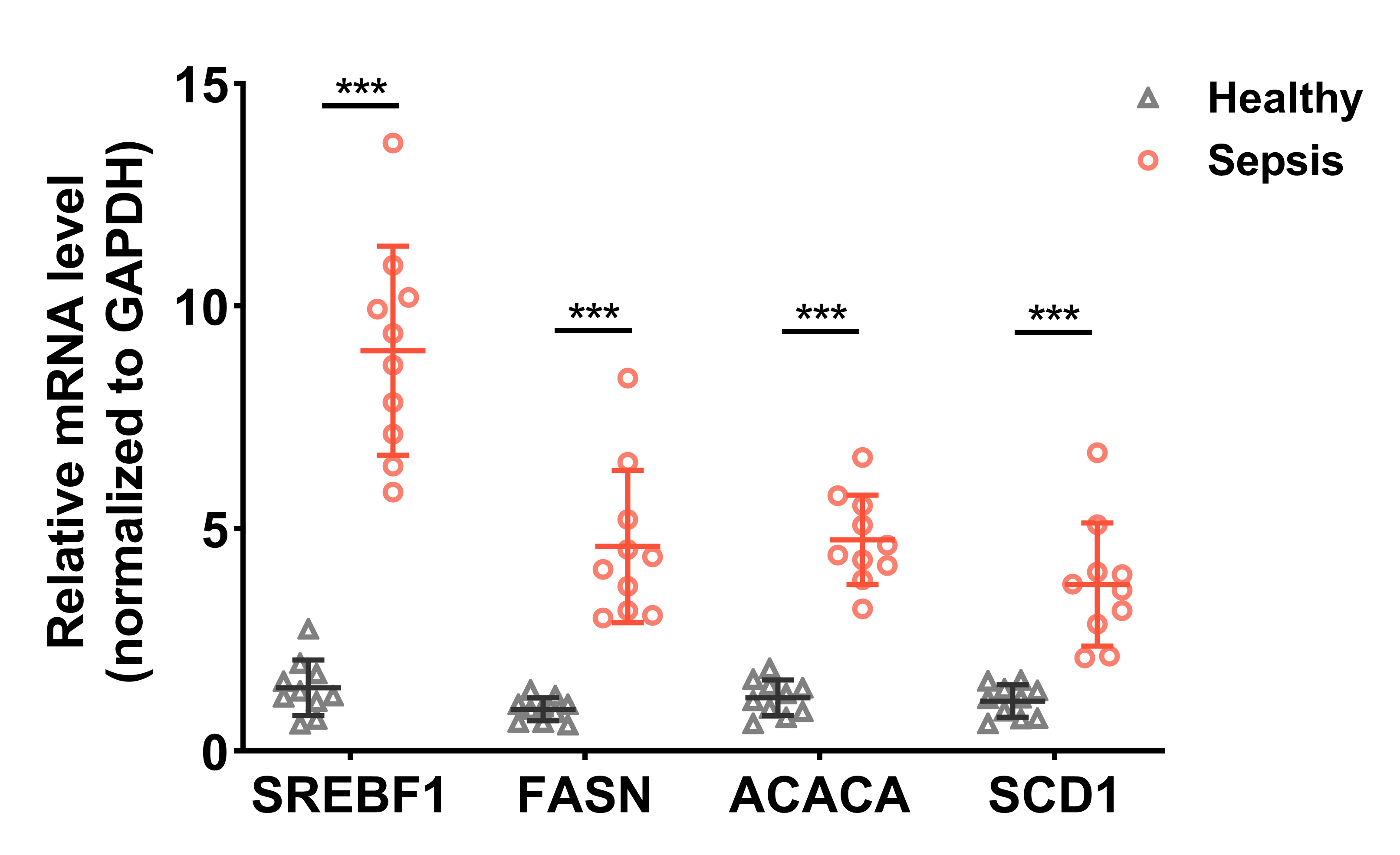


**Supplementary Figure 2.** Relative mRNA expression of lipid synthesis–related genes in PBMCs from healthy controls and sepsis patients.

mRNA levels of SREBF1, FASN, ACACA, and SCD1 were quantified in PBMCs isolated from healthy controls (*n* = 10; gray triangles) and sepsis patients (*n* = 10; red circles) using qPCR. Gene expression was normalized to GAPDH. Data are presented as mean ± SD; ***, *P* < 0.001.


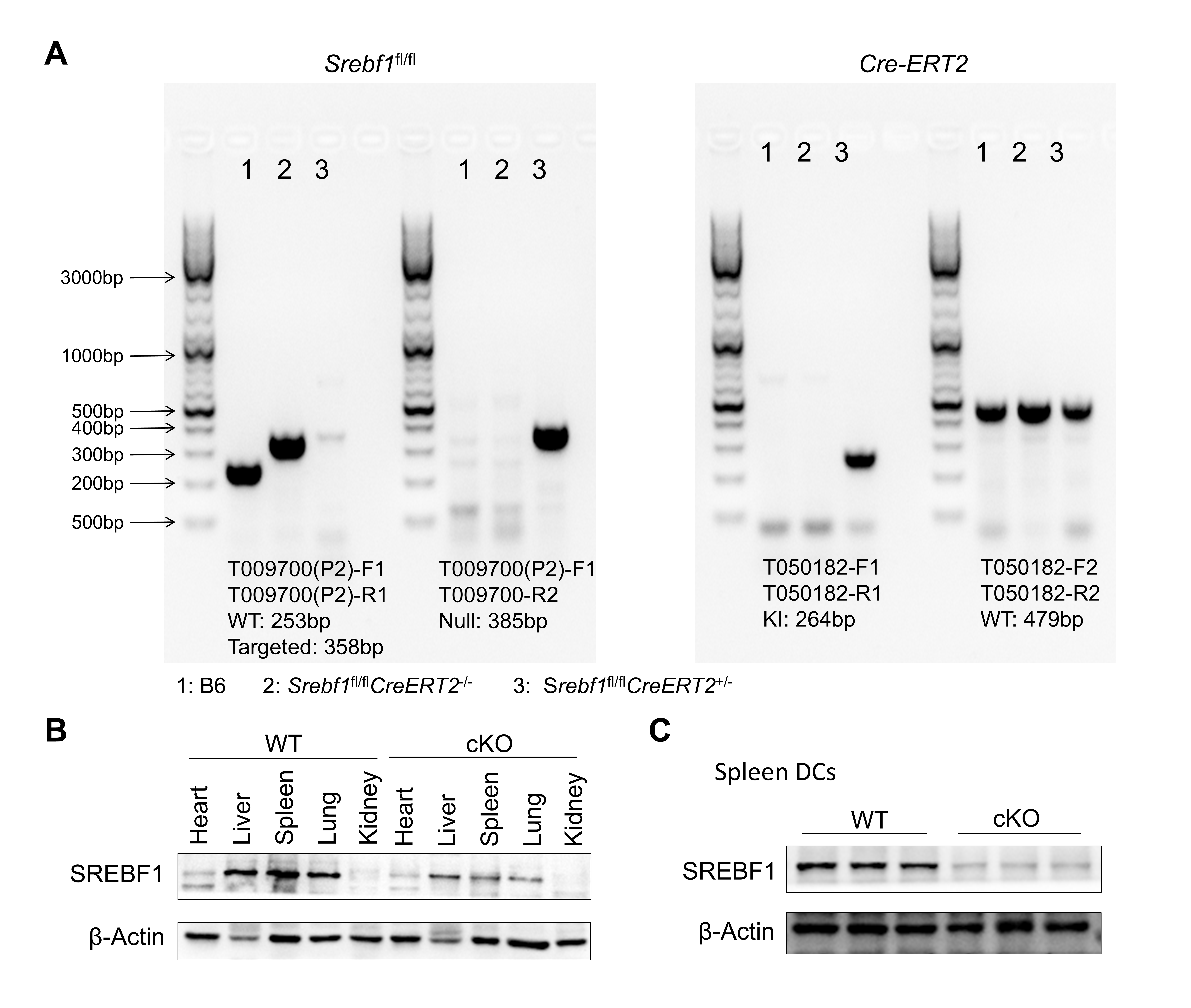


**Supplementary Figure 3.** Genotyping and validation of SREBF1 deletion in Srebf1 cKO mice.

(A) Agarose gel electrophoresis of genomic DNA isolated from the tails of WT, Srebf1^fl/fl^*CreERT2^-/-^*, and Srebf1^fl/fl^*CreERT2^+/-^* mice. (B-C) Western blots to confirm Srebf1 knockout in cKO mice.


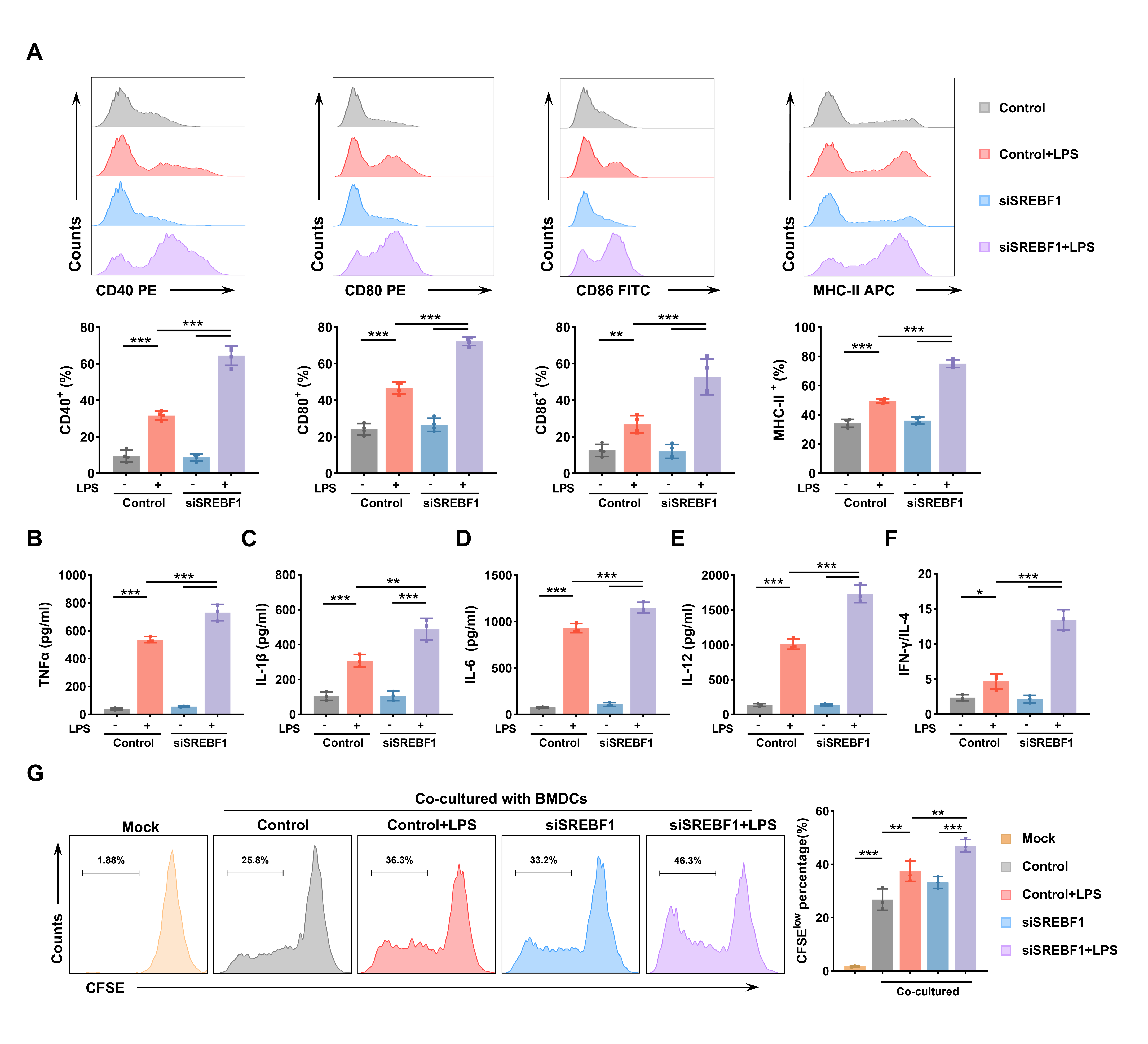


**Supplementary Figure 4.** Protective effect of SREBF1 silencing on DC immune function following LPS exposure.

(A) Flow cytometric analysis of co-stimulatory phenotypes expressed on control and siSREBF1 treated BMDCs with or without LPS (100 ng/mL) stimulation for 12 h (*n* = 3). (B–E) ELISA quantification of inflammatory cytokines released into the supernatant of BMDCs treated as described in (A) (*n* = 3). (F) CD4^+^ T cells were co-cultured with BMDCs (DC: T cell ratio = 1:100) treated as in (A). Levels of IFN-γ and IL-4 in the supernatant were measured by ELISA, and the IFN-γ/IL-4 ratio was calculated to assess Th1/Th2 polarization (*n* = 3). (G) The proliferative capacity of CD4^+^ T cells induced by BMDCs treated as in (A) was assessed using CFSE labeling and flow cytometry (*n* = 3). Data are presented as the mean ± SD; *, *P* < 0.05; **, *P* < 0.01; ***, *P* < 0.001. LPS, lipopolysaccharide; BMDCs, bone marrow-derived dendritic cells; CFSE, 5,6-carboxyfluorescein diacetate succinimidyl ester.


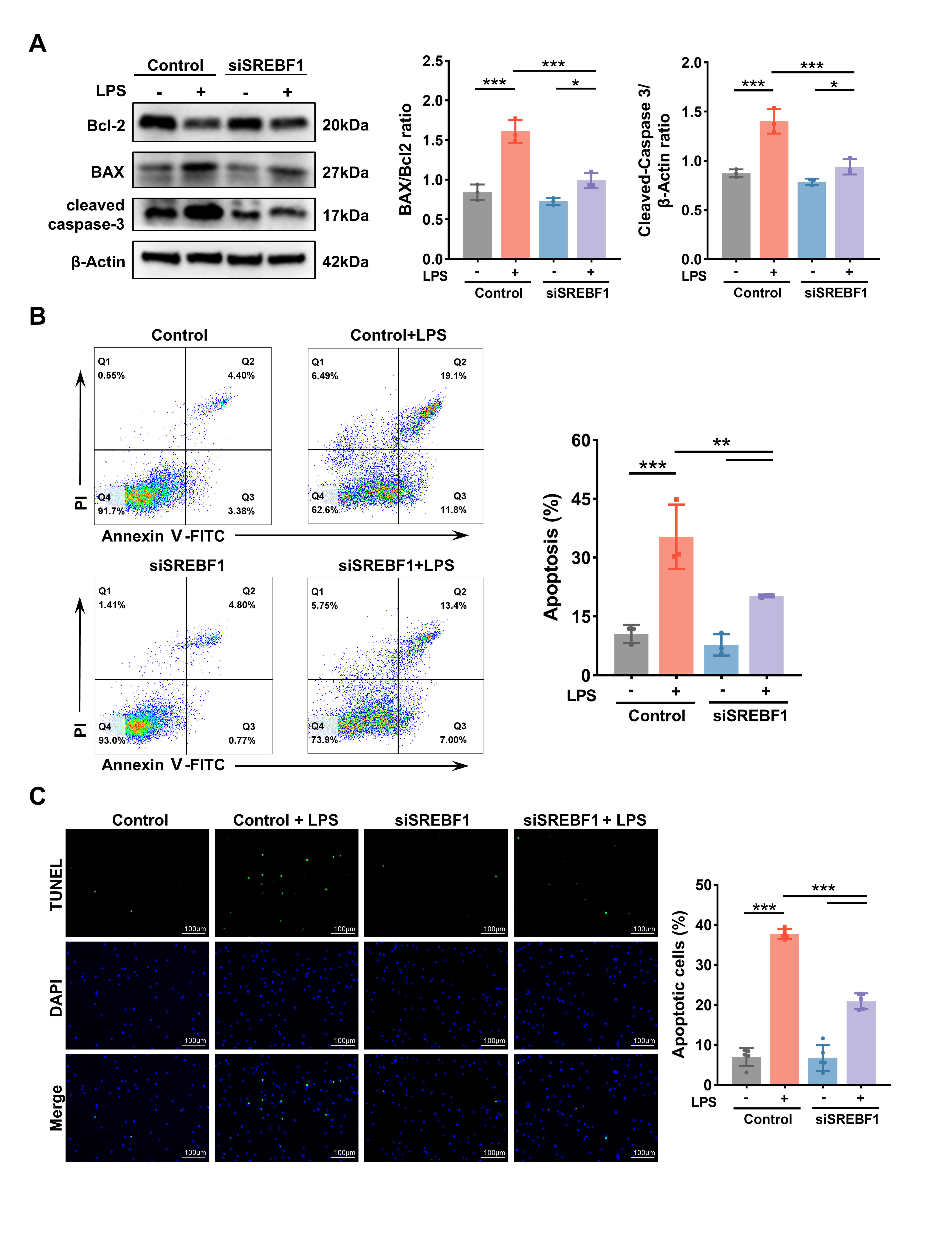


**Supplementary Figure 5.** SREBF1 silencing enhances the anti-apoptotic capacity of DCs after LPS.

(A) Immunoblot analysis of Bcl-2, BAX, and cleaved caspase-3 protein expression in control and siSREBF1-transfected BMDCs with/without LPS (100 ng/mL) stimulation for 12 h (*n* = 3). (B) Apoptosis rates of BMDCs treated as described in (A), assessed by flow cytometry (*n* = 3). (C) Representative fluorescence images of apoptotic cells stained with TUNEL in BMDCs treated as (A), along with statistical analysis of the percentage of apoptotic cells (*n* = 5). Scale bars, 100 μm. Data are presented as the mean ± SD; *, *P* < 0.05; **, *P* < 0.01; ***, *P* < 0.001. LPS, lipopolysaccharide; TM, tunicamycin.


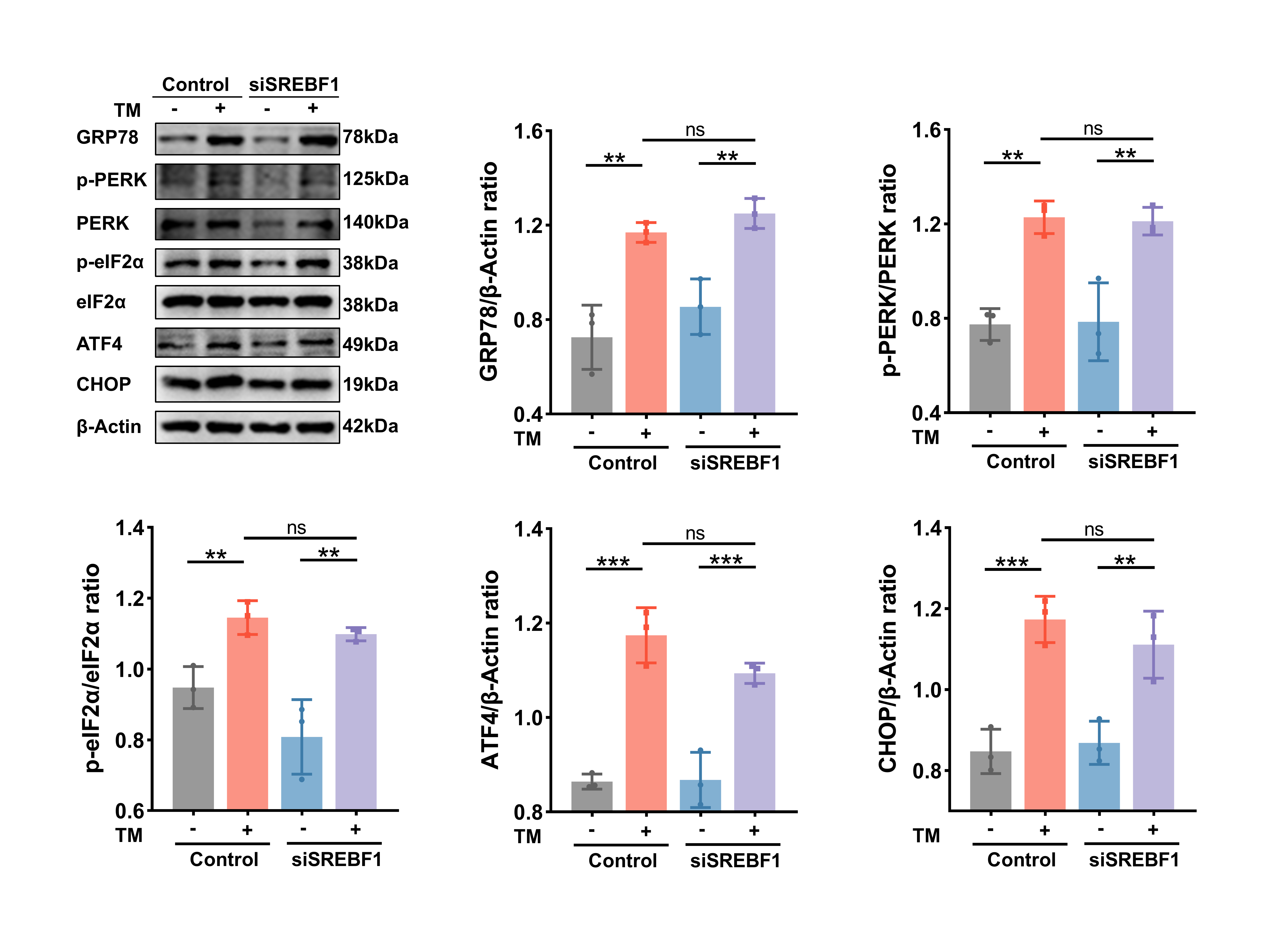


**Supplementary Figure 6.** Effects of SREBF1 silencing on TM-induced ER stress in BMDCs. Representative Western blots and quantitative analyses of GRP78, p-PERK, PERK, p-eIF2α, eIF2α, ATF4, and CHOP protein levels in control and SREBF1-silenced BMDCs treated with or without TM (0.5 μg/mL) for 12 h (*n* = 3). Data are presented as mean ± SD; ns, not significant, *, *P* < 0.05; **, *P* < 0.01; ***, *P* < 0.001. TM, tunicamycin.

**Supplementary Table 1.** Primer sequences for Real-time PCR

| Primer number | Primer name | Primer sequence | Product size |
| --- | --- | --- | --- |
| 1 | T009700(P2)-F1 | ATTTGGACTCAGTGAGACTAGGCCAG | WT: 253bp  Fl: 358bp |
|  | T009700(P2)-R1 | AGGGTCGTTCTAACGGCCTCTGA |  |
| 2 | T009700(P2)-F1 | ATTTGGACTCAGTGAGACTAGGCCAG | WT: 3628bp  Fl: 3849bp  Null: 385bp |
|  | T009700-R2 | CCCTGTGAATACTCACAACGGTGT |  |
| 3 | TO50182-F1 | CCCAAAGTCGCTCTGAGTTGTTA | KI: 264bp |
|  | TO50182-R1 | TTCCTCCTACATAGTTGGCAGTG |  |
| 4 | T050182-F2 | CCCAAAGTCGCTCTGAGTTGTTA | WT: 479bp |
|  | T050182-R2 | TCGGGTGAGCATGTCTTTAATCT |  |
| 5 | SREBF1-F | ACTGGTCGTAGATGCGGAGAAG |  |
|  | SREBF1-R | TGTCATTGATGGAGGAGCGGTAG |  |
| 6 | FASN-F | CCTGGCTGCTACTACATCG |  |
|  | FASN-R | CACATTCAAGGCCACGCA |  |
| 7 | ACACA-F | TAGTCTGCCACGGATCCAGA |  |
|  | ACACA-R | GGGAGGGATCTCTGAGGGTT |  |
| 8 | SCD1-F | CTTGCGATATGCTGTGGTGC |  |
|  | SCD1-R | CCGGGGCTAATGTGTCTTGT |  |
